# The Impact of Educating Parents of Leukemic Children on the Patients’ Quality of Life

**Published:** 2011-08-01

**Authors:** F Hashemi, N Asadi, N Beheshtipour, M Karimi

**Affiliations:** 1 Nursing and Midwifery College, Shiraz University of Medical Sciences, Shiraz, Iran; 2 Hematology Re-search Center, Nemazee Hospital, Shiraz University of Medical Sciences, Shiraz, Iran

**Keywords:** Leukemia, Children, Education, Parent, Iran

## Abstract

**Background:**

The quality of life of children with leukemia is reduced by fear and anxiety of parents after diagnosis, lack of information about the disease, treatments, and care of the child. This study aims to evaluate the effect of educating parents of leukemic children on the patients’ quality of life.

**Methods:**

In this interventional study, sixty parents of ALL children who met inclusion criteria were selected using simple random sampling method, and assigned to the experimental and control groups. The study tool included a valid and reliable questionnaire (TNO-AZL), that was filled in through interview by parents before and two months after the intervention for both groups. The first part of the questionnaire included demographic items and the second part (7 dimensions, each with 8 sections) contained questions related to the quality of life. The scores could range between 56 and 280 and a higher score represented a better quality of life. The intervention included three one-hour classes composed of lecture and question-answer sessions which were held for groups of 4-6 participants, accompanied by a booklet.

**Results:**

Before the intervention, the quality of life score in the experimental and control groups was 180.83±14.43 and 174.28±20.72, respectively; after the intervention, these figures changed to 226.9±11.76 and 174.41±20.42 respectively. Paired samples T-test proved a significant increase in the quality of life in the experimental group.

**Conclusion:**

Parent education successfully increased the quality of life of leukemic children; therefore, parental consultation sessions and educational programs are recommended.

## Introduction

Leukemia is the most common malignancy of children with a prevalence of 129 in one million, and the second cause of death among children aged 5 to 14 years. Acute lymphoblastic leukemia (ALL) is the most common type of this disease[1] accounting for 75% of all leukemias and 30% of all malignancies in childhood. ALL affects boys twice as much as girls.[2] In the United States of America, the incidence of ALL has risen from 1980 to 2002 and the current incidence rate is approximately 3-4 in 100’000 children under 15 years of age.[3] ALL is also the most common malignancy in children of south-western Iran with a prevalence of 44.5%[4] and has been among the 10 top prevalent cancer in Fars Province, Southern Iran.[5]

The survival rate of children with ALL has increased during the past 30 years, based on a study in Canada.[6] Occasionally, chemotherapy lasts for more than 3 years.[7] and today, increasing life expectancy and rehabilitating the patients to gain an appropriate quality of life are the cornerstones of therapeutic programs for children with hematological malignancies.[3] The concept of quality of life is generally considered as one’s perception of her/his welfare which originates from his/her current life experience.[8] Several studies have noted a decline in the quality of life in leukemic children.[9][10][11] Studies have also indicated that enhancement of the parents’ knowledge about the problems and needs of their leukemic children has an important effect on family support, leading to a significant increase in the quality of life of these children.[12][13] Other studies have confirmed that such interventions affect the quality of life of children with other chronic diseases.[14][15][16] Till now there was no research conducted in the impact of educating parents of leukemic children on the patients’ quality of life in Iran. This study aims to determine the effect of educating the parents of leukemic children on the patients’ quality of life.

## Materials and Methods

This interventional study was performed in Motahhari Clinic of Shiraz University of Medical Sciences and Afzalipoor Hospital of Kerman University of Medical Sciences in the southern regions of Iran in 2009. Sixty parents of ALL children were selected using simple random sampling, and assigned into the intervention and control groups (30 participants in each group). Inclusion criteria were parents with children with the diagnosis of ALL between 1 month and 2 years prior the present study, the leukemic child under maintenance therapy, no other chronic disease expect ALL, age range of 7-10 years, and children with standard ALL (i.e. a white blood cells count less than 50000/fL, absence of chromosomal anomalies, and documented response to therapy in the first month of treatment).[1] Also, children with parents and siblings without any accompanying disease were included. Their family type should be clear. The participating parents should be literate and should not have used other consultation systems previously. The parents were required to give an informed consent to join the study. The exclusion criteria were the participating child’s death, lack of interest of parents to cooperate in the study.

A previously validated reliable questionnaire (TNO-AZL: parent form) was used to assess the quality of life of the children before and two months after the intervention. This questionnaire was improved the first time by Vogels et al. in TNO Institute of Prevention and Health in Neherlands.[17][18] They conventionally called it TNO-AZL. The first part of the questionnaire included demographic items and the second part contained questions related to the quality of life in 7 dimensions {physical (body) complaints, autonomous (auto), social, cognitive and motor functioning, positive and negative emotions}. Each dimension was composed of 8 subgroups; each subgroup was scored between 1 and 5 on a Likert scale (representing the five levels “always”, “most of the time”, “sometimes in 2 recent weeks”, “rarely” and “never”). For positive emotions dimension, choosing “always” was scored 5 and choosing “never” was scored 1; for other dimensions, “always” was scored 1 and “never” was scored 5. The scores could range between 56 (a score of 8 for each dimension) and 280 (a score of 40 for each dimension) and the higher score represented a better quality of life.[17] Content validity of this questionnaire has been confirmed in studies by Landolt et al. in Germany and Soory et al. in Iran.[10][19] The reliability of the questionnaire was measured using Kronbach alpha and reported as 0.66-0.77 by Landolt et al. and 0.73 by Soori et al. [10][19]

The participating parents were assisted by one of the researchers to complete the questionnaire in the beginning of the study. A coding system was used to ensure that data collection was performed blindly. The intervention included a combination of three onehour classes composed of lecture and questionanswer sessions which were held for groups of 4-6 participants, each lasting for 45-60 minutes, and a booklet.[19] The first class focused on the nature of leukemia, basics of therapeutic approaches, effect of disease on the patient’s family, and coping strategies. The second class centered around techniques to communicate with the suffering child, effects of disease on various aspects of patient’s life, and solutions for increasing their quality of life. The third session was dedicated to patient care in hospital and at home. Depending on the educational level of the participating parents, learning-assistance tools such as posters were used to ensure that the learning material was completely comprehended by the participants. The participants could also review the contents of the sessions using the booklets.

Two months after the completion of interventions, the quality of life questionnaire was filled in again by the parents assisted by a researcher. The data were analyzed using statistical package for the Social Sciences (SPSS, version 11.5, Chicago, IL, USA) and a p-value less than 0.05 was considered as statistically significant. T and Chi-Square tests were used to compare results. This study was approved by the ethics committees of the affiliated universities.

## Results

The mean age of the mothers was 36.1 years in the experimental and 36.33 years in control group, and these figures for the fathers were 42.66 and 40.86 years. The mean age of children was 8.45 years in the experimental and 8.13 years in the control group. Seventy percent of the patients in the experimental and 80% in the control group were boys. While 53.3% of the fathers in the experimental and 46.7% in the control group worked in private section and 96.7% were unemployed housewives. The majority of fathers (73.3% in both arms) and mothers (76.7% in experimental and 80% in control group) were educated only to the primary school level. No difference was noticed in the aforementioned characteristics among the two arms of the study.

Before the intervention, the quality of life score in the experimental and control groups was 180.83±14.43 and 174.28±20.72, respectively; after the intervention, these figures changed to 226.9±11.76 and 174.41±20.42, respectively. A significant increase was seen in the quality of life in the experimental group (p<0.001, Table 1). Quality of life scores for each dimension before and after intervention, and betweengroup difference are compared in Table 2.

Scores increased in all dimensions after the intervention, with the maximal increase observed in negative emotions dimension (9.23±3.19) and the minimal change noted in motor functioning dimension (3.33±1.53). According to the results, there was no change in the control group scores for negative emotions dimension (0/00±0/45), and the score given to autonomous and motor functioning decreased over time (-0/03±0/61 and -0/06±2/82). The change of quality of life of score before and after 2 months intervention in the experimental group was 46.06±10.26 and in the control group it was 0.13±1.37. Paired ttest confirmed a significant augmentation in the quality of life scores in all dimensions in the intervention group (p<0.001). However, the changes in the quality of life scores in the control group were not significant. A significant difference was visible in change of quality of life of score before and after 2 months intervention in the experimental group (both of all seven dimensions and total score) (p<0.001).

**Table 1:** Comparison of the quality of life scores before and after intervention in the two study groups.

**Time**	**Before intervention mean±standard deviation**		**P value a**	**After intervention mean±standard deviation**		**P value a**
**Mean score Dimensions of Quality of life**	**Experiment**	**Control**		**Experiment**	**Control**	
Physical (Body) complaints	25.66 ± 4.89	23.5 ± 5	0.09	31.83 ± 3.47	23.53 ± 4.74	< 0.001
Autonomous functioning	28.26 ± 4.75	28.26 ± 5.21	0.95	34.7 ± 3.30	28.20 ± 5.35	< 0.001
Social functioning	24.36 t 4.01	24.2 ± 3.80	0.92	31.1 ± 3.40	24.26 ± 3.67	< 0.001
Motor functioning	30.53 ± 2.60	30.16 ± 3.71	0.63	33.86 ± 2.04	30.13 ± 3.64	< 0.001
Cognitive functioning	25.36 ± 5.18	24.50 ± 3.70	0.45	31.13 ± 3.85	24.53 ± 3.71	< 0.001
Positive emotions	26.26 ± 3.99	24.36 ± 3.69	0.07	33.96 ± 2.20	24.46 ± 3.73	< 0.001
Negative emotions	20.76 ± 3.29	19.30 ± 4.38	0.14	30 ± 2.66	19.30 ± 4.53	< 0.001
Total score	180.83 ± 14.43	174.28 ± 20.72	0.16	226.9 ± 11.76	174.41 ± 20.42	< 0.001

^a^ Independent two-sample T-test

**Table 2:** Comparison of quality of life scores and the change of scores before and after intervention in the two study groups

**Group**	**Experimental mean±standard deviation**		**P Value a**	** Control mean±standard deviation**		**P Value a**	**Change of score mean±standard deviation**		**P Value b**
**Dimensions mean score of quality of life mean**	**Before**	**After**		**Before**	**After**		**Experiment**	**Control**	
Physical complaints	25.66 ± 4.89	31.83 ± 3.47	< 0.001	23.5 ± 5	23.53 ± 4.74	0.80	6.16 ± 2.73	0.53 ± 2.82	< 0.001
Autonomous functioning	28.26 ± 4.75	34.7 ± 3.30	< 0.001	28.26 ± 5.21	28.20 ± 5.35	0.09	6.43 ± 3.23	-0.06 ± 2.82	< 0.001
Social functioning	24.36 ± 4.01	31.1 ± 3.40	< 0.001	24.2 ± 3.80	24.26 ± 3.67	0.44	6.73 ± 2.71	0.06 ± 1.33	< 0.001
Motor functioning	30.53 ± 2.60	33.86 ± 2.04	< 0.001	30.16 ± 3.71	30.13 ± 3.64	0.57	3.33 ± 1.53	-0.03 ± 0.61	< 0.001
Cognitive functioning	25.36 ± 5.18	31.13 ± 3.85	< 0.001	24.50 ± 3.70	24.53 ± 3.71	0.32	5.76 ± 2.84	0.20 ± 0.80	< 0.001
Positive emotions	26.26 ± 3.90	33.96 ± 2.20	< 0.001	24.36 ± 3.69	24.46 ± 3.73	0.08	7.70 ± 3.01	0.10 ± 0.95	< 0.001
Negative emotions	20.76 ± 3.29	30 ± 2.66	< 0.001	19.30 ± 4.38	19.30 ± 4.53	1.00	9.23 ± 3.19	0.00 ± 0.45	< 0.001
Total score	180.83 ± 14.43	226.9 ± 11.76	< 0.001	174.28 ± 20.72	174.41 ± 20.42	0.39	46.06 ± 10.26	0.13 ± 1.37	< 0.001

^a^ Paired two-sample T-test

^b^ Independent two-sample T-test

## Discussion

At the baseline, there was no difference in the quality of life scores, and both groups received a moderate score in all dimensions. This is congruent with the baseline scores reported by Landolt et al. study in Germany, Santos et al. Study in Brazil, and Redalli et al. in Italy.[11][20][21] Speechly et al. has also reported a lower quality of life score for leukemic children compared to analogous healthy children.[6] This necessitates interventions which are oriented towards a better quality of life.

Our findings indicate a higher quality of life in children two months after the intervention is given to their parents, in all of the seven measured dimensions (p<0/001). In another study in Iran, Allahyari et al. noted a significant increase in the quality of life of children with thalassemia after the effect of family – centered improvement Model on the Quality of life of school-age B-thallasemic children.[15] Iconomou et al. have reported similar outcomes after a booklet containing information about chemotherapy was given to Greek adult patients with malignant diseases.[22] Our findings are also in accordance with the outcomes of Lorenzo et al.’s study in Italy. They showed that a combination of lectures, booklets and video films improved the quality of life of adult patients with malignant diseases after their first round of chemotherapy.[19]

We noted the largest score increase in the negative emotions dimension and the least increase in motor functioning. This implies that our educational intervention for parents has effectively reduced such negative emotions in the leukemic children as sadness, aggressiveness, anger, restlessness, jealousy, depressed mood and anxiety, while having only a minimum effect on motor functioning like running, walking, playing, etc. This is also possible that we observed little changes in motor functioning because of the short time-span of our study (two months) or because children undergoing chemotherapy have fewer chances to improve in terms of the quality and quantity of motor functioning (that is, motor functioning further improve after the completion of the course of chemotherapy and resolution of its side effects). Nevertheless, improvement in scores given in all seven dimensions was statistically significant (p<0.001).

Based on the information in Table 2, a slight increase in quality of life scores in physical complaints, social and cognitive functioning and positive emotions dimensions in the control group although it was not statistically significant. This can be attributed to the fact that parents in the control group could also gain information via informal sources during the study time-span, including their contact with physicians and parents in the experimental group who were involved in the current study or through the experience gained by children, and their parents by coping with the critical circumstances related to the disease. There was no change in the control group scores for negative emotions dimension, and the score given to autonomy and motor functioning decreased over time .This might be due to the nature of the disease and its side effects along with the prolonged courses of treatment repeated chemotherapy sessions, and lack of formal education in the parents of the control group.

A related research conducted by Barrera et al. aimed to evaluate the role of social support in the emotional coping of siblings of children with malignant diseases. Their study showed that the siblings of children with malignant diseases who benefitted from higher social support presented signs and symptoms of anxiety, depression or behavioral problems less frequently. This is in the same line with the results of the present study.[23] Also, improvement of social activities in the case group of the present study resembles to that of Sidhu et al. In the Sidhu et al.’s study, social competency of siblings of children with cancer was improved significantly after conducting a peer support camp for them.[24] Furthermore, the results of the study by Packman et al. on the effect of psychologic interventions on the signs and symptoms of stress disorder, anxiety, self-esteem and quality of life of siblings of children with cancer indicated improvement in the aforementioned self-esteem and quality of life and a decrease in stress and anxiety symptoms.[25] These are all consistent with the results of current study, which signifies an improved quality of life in leukemic children after educational intervention in their parents. Our findings are also consistent with the results of another study by Golchin et al. which proves the positive effect of educational and self-control programs on the quality of life of adult patients with leukemia.[26]

In short, our findings indicate that parent education successfully increased the quality of life of leukemic children further than potential gain of similar information from informal sources. Parent education leads to better understanding of Leukemia, chemotherapy and their side effects, as well as the negative impact of this disease on children’s quality of life,[27] which in turn results in improved function of parents in terms of communicating with the suffering child, providing appropriate care, and facing related problems. This improvement in the function of parents yields a higher quality of life in the leukemic children.

Effective planning of educational interventions for parents, leads to lower stress and higher quality of life in children with leukemia. This study explains the stipulation of educating parents about the effects of leukemia on the quality of life of the affected children and their families, and appropriate methods to face the consequential problems. Members of therapy team, especially nurses, should be trained in this regard to be able to meet the requirements of these parents. We recommend setting up consultation clinics in the centers providing health-care to leukemic children, where nurses offer education to parents of these children and enable them to face and resolve ensuing problems independently. In this way, the problems of such patients will be prevented, having an important effect on the quality of life of these patients.
